# Combination of C-reactive protein/albumin ratio and time to castration resistance enhances prediction of prognosis for patients with metastatic castration-resistant prostate cancer

**DOI:** 10.3389/fonc.2023.1162820

**Published:** 2023-06-02

**Authors:** Yozo Mitsui, Fumito Yamabe, Shunsuke Hori, Masato Uetani, Hiroshi Aoki, Kei Sakurabayashi, Mizuho Okawa, Hideyuki Kobayashi, Koichi Nagao, Koichi Nakajima

**Affiliations:** Department of Urology, Toho University Faculty of Medicine, Tokyo, Japan

**Keywords:** C-reactive protein albumin ratio, time to castration resistance, metastatic castration-resistant prostate cancer (mCRPC), metastatic hormone sensitive prostate cancer, biomarker

## Abstract

**Objective:**

This study aimed to identify the prediction accuracy of the combination of C-reactive protein (CRP) albumin ratio (CAR) and time to castration resistance (TTCR) for overall survival (OS) following development of metastatic castration-resistant prostate cancer (mCRPC).

**Methods:**

Clinical data from 98 mCRPC patients treated at our institution from 2009 to 2021 were retrospectively evaluated. Optimal cutoff values for CAR and TTCR to predict lethality were generated by use of a receiver operating curve and Youden’s index. The Kaplan–Meier method and Cox proportional hazard regression models for OS were used to analyze the prognostic capabilities of CAR and TTCR. Multiple multivariate Cox models were then constructed based on univariate analysis and their accuracy was validated using the concordance index.

**Results:**

The optimal cutoff values for CAR at the time of mCRPC diagnosis and TTCR were 0.48 and 12 months, respectively. Kaplan–Meier curves indicated that patients with CAR >0.48 or TTCR <12 months had a significantly worse OS (both *p* < 0.005). Univariate analysis also identified age, hemoglobin, CRP, and performance status as candidate prognostic factors. Furthermore, a multivariate analysis model incorporating those factors and excluding CRP showed CAR and TTCR to be independent prognostic factors. This model had better prognostic accuracy as compared with that containing CRP instead of CAR. The results showed effective stratification of mCRPC patients in terms of OS based on CAR and TTCR (*p* < 0.0001).

**Conclusion:**

Although further investigation is required, CAR and TTCR used in combination may more accurately predict mCRPC patient prognosis.

## Introduction

Prostate cancer (PC) is the most common type of cancer in men and the second leading cause of cancer-related death worldwide (1). In Japan, PC has the highest prevalence of all male cancers, with 94,748 newly diagnosed cases reported in 2019 (2). Metastatic hormone-sensitive PC (mHSPC) at the initial diagnosis accounts for approximately 4% of all PC cases, with the main systemic therapy commonly given androgen deprivation therapy (ADT), as this cancer type grows in an androgen-dependent manner (3, 4). However, response to ADT by metastatic PC is usually temporary and cancer relapse occurs within 6 months to several years in a large number of patients, leading to metastatic castration-resistant PC (mCRPC).

mCRPC is an advanced condition and with a poor prognosis. When treating affected patients, the ability to predict treatment outcome and life prognosis plays important roles for distinguishing those who may benefit from treatment and avoiding unnecessary adverse effects. Factors, such as the original biological characteristics of the tumor, or genomic alterations in cancer cells and selective survival of highly resistant subclones induced by ADT, have been found to be associated with acquisition of castration resistance in PC cases (5, 6). Nevertheless, the degree of involvement of such factors, type and number of therapeutic drugs available, and necessary treatment period until castration differ among individual cases; thus, mCRPC patients are considered to be a heterogeneous population. It is necessary to comprehensively evaluate factors such as tumor and host environment, and treatment course to accurately predict prognosis.

Serum C-reactive protein (CRP) and albumin levels are representative of chronic inflammation and nutritional status in cancer patients (7, 8). Chronic inflammation is thought to promote tumor progression by influencing the tumor environment, while the tumor itself can also induce inflammation, leading to progression and malignancy (9). In cancer patients, nutritional status deteriorates with progression due to inadequate nutrient intake and tumor overconsumption, resulting in hypoalbuminemia that stimulates various inflammatory cytokines, including interleukin 6, thus promoting CRP production in the liver (10). Therefore, serum CRP and albumin are considered as interrelated serum biomarkers that may reflect host and cancer status, respectively. Indeed, CRP albumin ratio (CAR), consisting of CRP and albumin, has been confirmed as a useful prognostic factor in many cancer types, including gastrointestinal (11–13), lung (14), and urological such as renal cell carcinoma (15). In addition, CAR has potential application for predicting prognosis of mCRPC cases (15–17).

Studies have shown that shorter time to castration resistance (TTCR) is associated with worse overall survival (OS) in PC patients following the initial diagnosis as well as after acquiring castration resistance (17–20). Wenzel et al. (20) speculated that duration of treatment response before PC becomes castration-resistant may be related not only to patient or baseline tumor characteristics, but also to genetic differences or gene mutations occurring in the host or tumor.

Thus, CAR and TTCR reflect prognostic characteristics of mCRPC patients from different aspects, and are speculated to have a mutually complementary relationship. This study investigated whether those in combination could be used to predict prognosis of mCRPC patients with higher accuracy than methods presently available.

## Materials and methods

### Patients and treatments

The records of 159 PC patients with castration resistance after receiving ADT plus bicalutamide and subsequent first-line treatment at our institution between 1 September 2009 and 31 November 2021 were retrospectively reviewed. After excluding 61 without metastasis at the time of castration resistance acquisition (60 non-meta HSPC cases and 1 mHSPC case at initial PC diagnosis), 98 mCRPC patients were enrolled. As first-line treatment for mCRPC, each received androgen receptor axis-targeted therapy (ARAT) using either enzalutamide or abiraterone, as well as first-generation antiandrogens (AAs) including flutamide and estramustine, docetaxel (DTX), or radium-223 (Ra-223). Therapy was continued until disease progression, occurrence of an unacceptable adverse event, or patient refusal. Since July 2014, ARAT has been available for mCRPC at our institution and 25 of the present patients who started treatment before that time did not have that as a first-line option, though most had ARAT available for a subsequent treatment course.

For this retrospective study, patient consent was not required, though information was posted on the hospital website indicating how to request exclusion. This study was conducted in accordance with the Declaration of Helsinki after receiving approval from the Ethics Committee of Toho University Omori Medical Center (no. M22168).

### Assessments

Patient characteristics at the time of PC diagnosis [serum prostate-specific antigen (PSA) level, Gleason score (GS), and metastatic sites] and start of first-line treatment for mCRPC, including age, body mass index, Eastern Cooperative Oncology Group performance status (PS), chemistry profile, levels of serum hemoglobin, white blood cells, lactate dihydrogen, alkaline phosphatase, total protein, albumin, CRP, and PSA, metastatic sites, and history of treatment with ARAT or DTX, were collected and assessed respectively. CAR was calculated from CRP and albumin values using the following formula: CRP (mg/L)/albumin (g/dl).

mCRPC was defined as serum testosterone level <50 ng/dl and either of the following factors present: (i) PSA value determined at intervals of 4 weeks increased by ≥25% from the lowest value, and with increase ≥2.0 ng/ml; or (ii) radiographic findings showing progression or appearance of new lesions (21). TTCR was defined as duration from beginning ADT treatment in mHSPC patients to first stated date of mCRPC. Serum PSA levels were measured every 4 weeks during treatment. PSA response after first-line treatment for mCRPC was defined as ≥50% reduction from pretreatment baseline. PSA progression was defined as three consecutive increases in that level of ≥50% over the nadir value at a minimum of 4.0 ng/ml.

The primary and secondary endpoints of the study were overall survival (OS) after development of mCRPC and time to PSA progression, respectively. For OS analysis, duration from beginning treatment for mCRPC to patient death during any course was used. Time to PSA progression was calculated from day of mCRPC diagnosis to final day of the study or evidence of progressive disease.

### Statistical analyses

**Figure 1:**
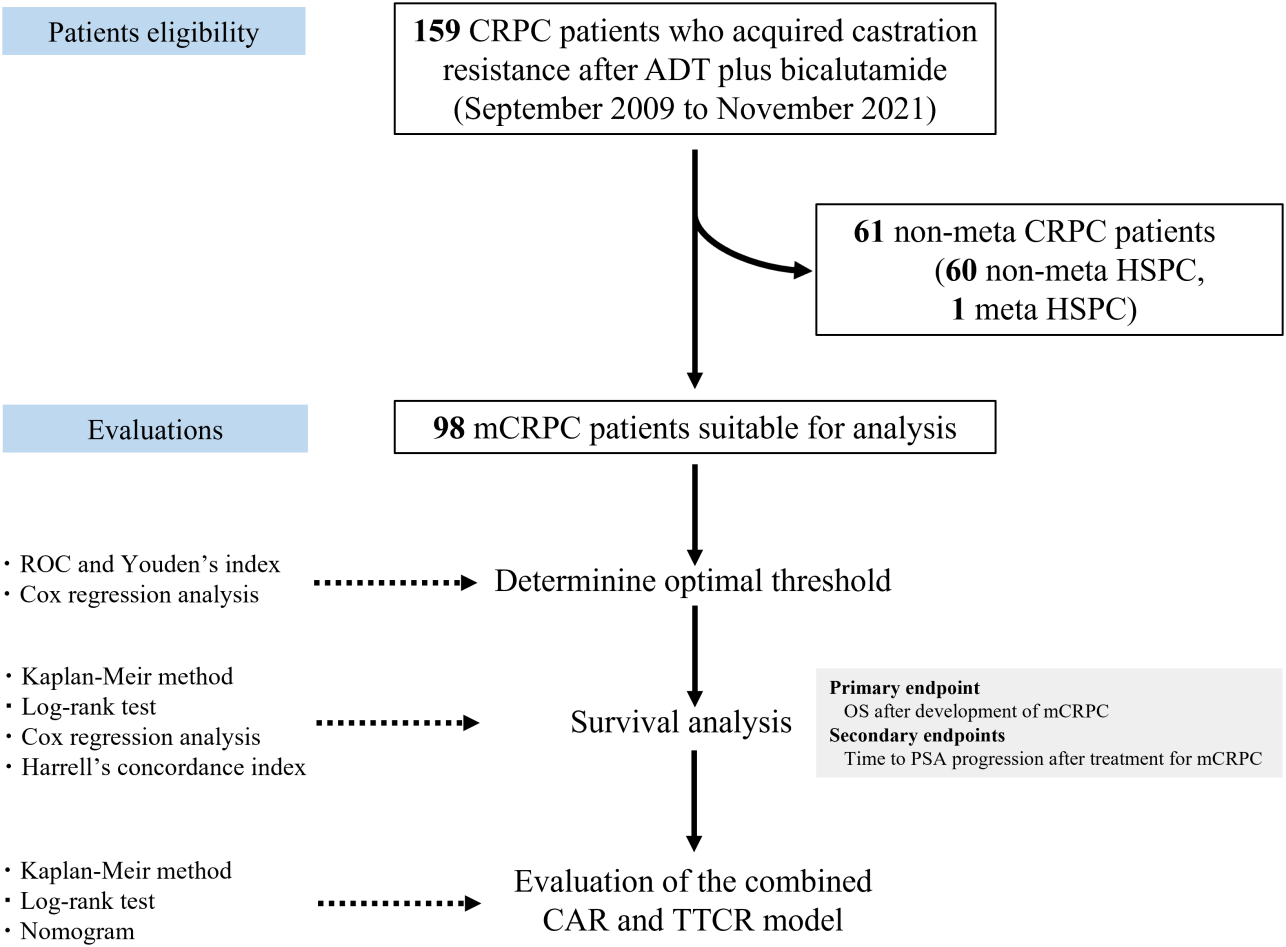
Flowchart showing patient eligibility, study design, and statistical methods. CRPC, castration-resistant prostate cancer; mCRPC, metastatic CRPC; ADT, androgen deprivation therapy; HSPC, hormone-sensitive prostate cancer; CAR, C-reactive protein albumin ratio; TTCR, time to castration resistance; ROC, receiver operating curve; OS, overall survival; PSA, prostate-specific antigen.

Measurement values are expressed as median (interquartile range; IQR), mean ± standard deviation (SD), or number (percent of total). Receiver operating characteristic (ROC) curve and Youden’s index values for both CAR and TTCR for predicting lethality were used to determine optimum threshold. The cohort was divided into three groups based on CAR and TTCR risk, then ANOVA or chi-square test results were used to analyze differences in characteristics among them. For evaluation of non-normal distributed continuous variables among the groups, a Kruskal–Wallis test was used. Survival curves were created using the Kaplan–Meier method and differences between them were analyzed with a log-rank test. Univariate analysis for OS was performed using a Cox proportional hazards regression model, followed by construction of two multivariate Cox models for OS based on univariate analysis, with accuracy validated by Harrell’s concordance index (C-index). A simple nomogram for predicting mCRPC prognosis was developed using the R “survival” package. *p*-values less than 0.05 were considered to indicate statistical significance. All data were analyzed using the statistical software application EZR (Easy R) (http://www.jichi.ac.jp/saitama-sct/SaitamaHP.files/statmed.html) (22). A flowchart showing determination of patient eligibility, study design, and statistical methods is presented in **Figure 1**.

## Results

### Patient characteristics

Clinicopathological characteristics of all 98 mCRPC patients are summarized in **Table 1**. Median follow-up duration from first mCRPC treatment was 28 months. Mean age at mCRPC diagnosis was 75.3 ± 8.8 years and body mass index was 22.3 ± 83.5 kg/m^2^. Forty-three (43.9%) had a PS of 0, and the remaining 55 (56.1%) had a score of 1 or 2 prior to starting first-line treatment. Among blood markers at treatment initiation, mean hemoglobin and albumin levels were 4.1 ± 0.5 and 12.4 ± 1.8 g/dl, respectively; median CRP level was 1.0 mg/L (0–2.0 mg/L) and mean CAR was 0.23 (0–0.59). At the time of mHSPC diagnosis, 19 patients (19.4%) were stage cT4, 48 (49%) had GS 9 or higher, and 74 (75.5%) had high-volume metastatic burden according to the CHAARTED criteria (23). Bone was the most common site of distant metastasis in 88 (89.8%) and visceral metastasis was found in 26 (26.5%). The major sites of visceral metastasis were lung in 12, paraaortic lymph node in 6, and liver in 2 cases. Median TTCR was 13.8 months (8.4 to 23.7 months). Initial therapy for mCRPC was ARAT in 50 (51.0%), first-generation AA in 37 (37.8%), DTX in 9 (9.2%), and Ra-223 in 2 (2.0%). During the study observation period, 90 (91.8%) were treated with ARAT and 42 (42.9%) were treated with DTX in either treatment course.

**Table 1:** Clinicopathological characteristics of 98 mCRPC patients.

Characteristics	
Age at mCRPC diagnosis, years	75.3 ± 8.8
Body mass index, kg/m^2^	22.3 ± 3.5
ECOG PS	
0	43 (43.9)
≥1	55 (56.1)
Serum markers at initial PC diagnosis	
PSA levels, ng/ml	188.0 (32.2–523.6)
Serum markers at mCRPC diagnosis	
PSA levels, ng/ml	9.5 (2.5–28.2)
Hemoglobin, g/dl	12.4 ± 1.8
White blood cell, ×10^9^/L	6.1 ± 2.0
Lactate dehydrogenase, U/L	222 (198–260)
Alkaline phosphatase, U/L	266 (208–404)
Total protein, g/dl	7.4 ± 0.6
Albumin, g/dl	4.1 ± 0.5
CRP, mg/L	1.0 (0–2.0)
CAR	0.23 (0–0.59)
Clinical T stage	
≤T3	79 (80.6)
T4	19 (19.4)
Gleason score	
≤8	50 (51.0)
≥9	48 (49.0)
Tumor burden at PC diagnosis (CHAARTED)	
High	74 (75.5)
Low	24 (24.5)
Regional lymph node metastasis at mCRPC diagnosis	48 (49.0)
Distant metastasis at mCRPC diagnosis	
Bone (total)	88 (89.8)
Bone (≥4)	67 (68.4)
Any viscera (lung, liver, etc.)	26 (26.5)
Time to castration resistance, months	13.8 (8.4–23.7)
First-line treatment for mCRPC	
ARAT	50 (51.0)
First-generation AAs	37 (37.8)
Docetaxel	9 (9.2)
Radium-223	2 (2.0)
Implementation of ARAT during treatment period	90 (91.8)
Implementation of docetaxel treatment during treatment period	42 (42.9)

Data are presented as median (interquartile range), mean ± standard deviation, or number (percentage). mCRPC, metastatic castration-resistant prostate cancer; ECOG PS, Eastern Cooperative-Oncology Group Performance Status Scale; CRP, C-reactive protein; CAR, CRP/albumin ratio; PSA, prostate-specific antigen; ARAT, androgen receptor axis-targeted therapy; AAs; antiandrogens.

### Evaluations of CAR and TTCR as prognostic factors

**Figure 2:**
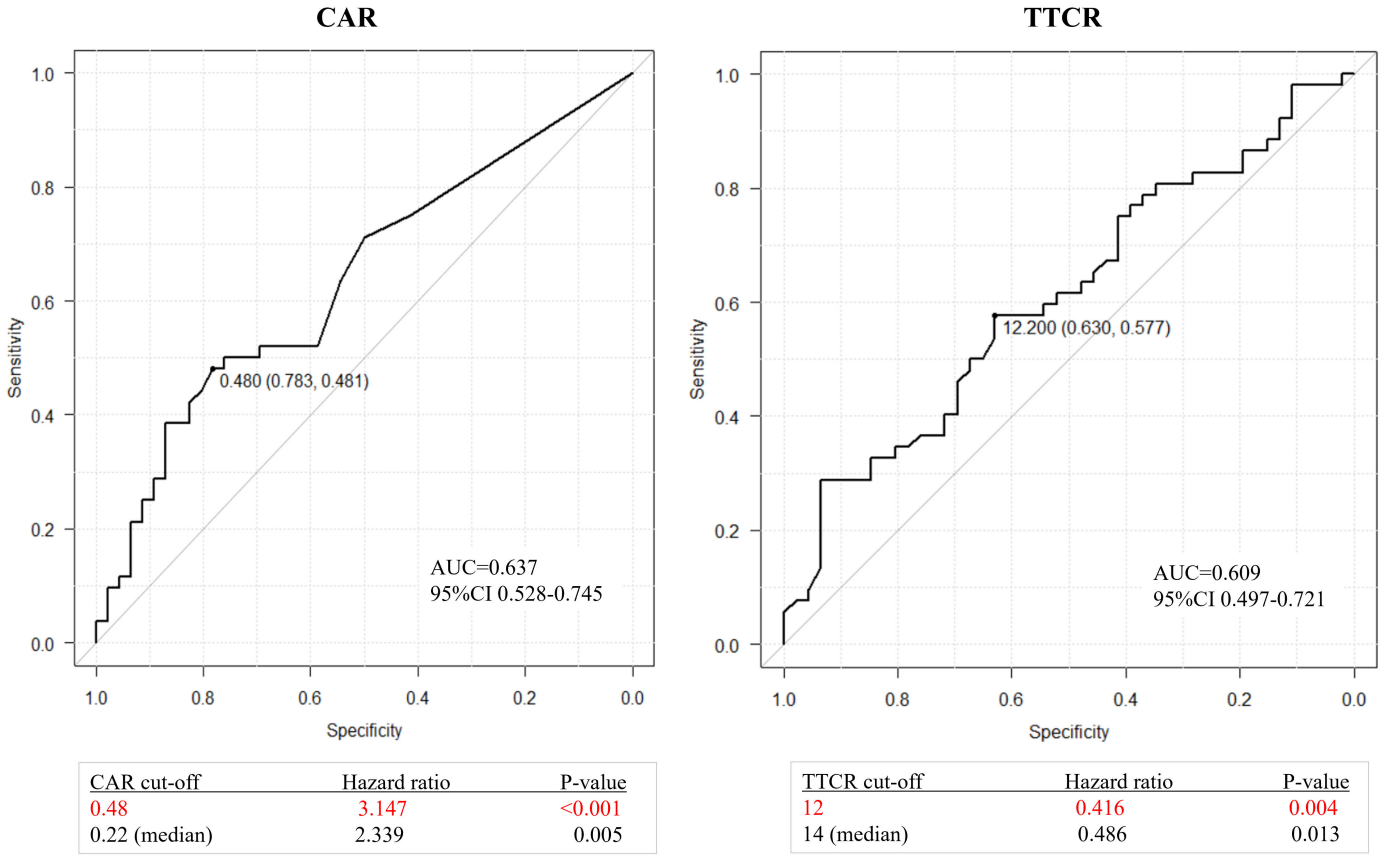
ROC curves for overall survival after castration resistance shown by CAR (CRP/Alb ratio) or TTCR (time to castration resistance). Optimal cutoff values for CAR and TTCR were determined to be 0.48 (area under the curve 0.637, sensitivity 0.481, and specificity 0.783) and 12.2 months (area under the curve 0.609, sensitivity 0.577, and specificity 0.630), respectively. Comparisons of these values with the cutoff value defined by the median confirmed the superiority of values determined with use of the Youden index.

**Figure 3:**
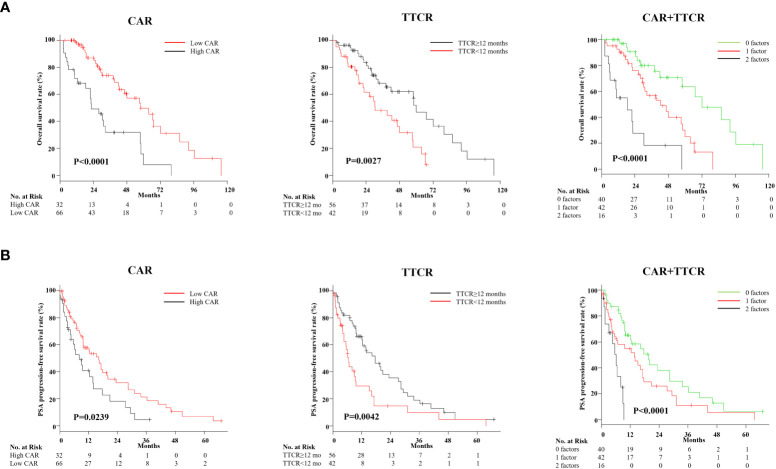
Kaplan–Meier analysis of overall survival after castration resistance, and PSA progression-free survival following first-line treatment for mCRPC based on CAR (CRP/Alb ratio) and TTCR (time to castration resistance). **(A)** Kaplan–Meier curves for OS based on CAR and TTCR, and those in combination. OS for the high CAR and TTCR <12-month groups was significantly worse than for the low CAR and TTCR ≥12-month groups, respectively. Risk stratification according to values for CAR and TTCR effectively stratified the prognosis of mCRPC patients. **(B)** Kaplan–Meier curves for PSA progression-free rate after first-line treatment against mCRPC based on CAR and TTCR, and those in combination. Similar to the results seen in the OS analysis, CAR, TTCR, and the combination of both factors provided correct risk classification regarding the duration of first-line treatment response.

Optimal cutoff values of CAR and TTCR for lethality prediction in mCRPC patients were examined. ROC curve analysis using Youden’s index revealed an optimal cutoff value of CAR for prediction of lethality of 0.48 (area under the curve 0.637, sensitivity 0.481, and specificity 0.783), while that of TTCR was 12.2 months (area under the curve 0.609, sensitivity 0.577, and specificity 0.630) (**Figure 2**). Using Cox analysis, these values were compared with the cutoff value defined by the median and the results confirmed that the hazard ratio (HR) for both values was superior as compared to the median value. Using these cutoff levels, patients were divided into low (≤0.48, *n* = 66) and high (>0.48, *n* = 32) CAR groups, and TTCR ≥ 12-month (*n* = 56) and TTCR < 12-month (*n* = 42) groups. Kaplan–Meier curve analysis showed that the high CAR group had significantly worse OS than the low CAR group (median 22.2 vs. 30.0 months, *p* < 0.0001) (**Figure 3A**). Similarly, the TTCR < 12-month group had worse OS than the TTCR ≥ 12-month group (median 20.7 vs. 30.0 months, *p* = 0.0027). Furthermore, a significantly shorter time to PSA progression was observed in patients with high CAR as compared to those with low CAR, as well as for the TTCR < 12-month as compared with the TTCR ≥ 12-month group (*p* = 0.0239 and *p* = 0.0042, respectively) (**Figure 3B**).

Uni- and multivariate Cox analyses for OS were performed to further evaluate CAR and TTCR prognostic value. Univariate analysis revealed that both CAR (HR 3.147, 95% CI 1.768–5.602, *p* < 0.0001) and TTCR (HR 0.416, 95% CI 0.230–0.750, *p* = 0.0036) significantly associated with OS (**Table 2**). Similarly, age (HR 2.135, 95% CI 1.072–4.252, *p* = 0.0309), ECOG PS (HR 2.318, 95% CI 1.288–4.174, *p* = 0.0051), hemoglobin level (HR 0.369, 95% CI 0.205–0.663, *p* = 0.0001), and CRP level (HR 2.459, 95% CI 1.405–4.304, *p* = 0.0016) were shown as candidate factors for a significant association with OS (**Table 2**). To avoid the influence of possible multicollinearity between CAR and CRP, two multivariate Cox proportional hazard models based on the same four candidate factors (TTCR, ECOG PS, age, and hemoglobin), which exhibited a significant association in univariate analyses, and CAR or CRP were constructed. The C-index for model I with CAR was 0.757, higher than the value for model II with CRP (0.746) in terms of OS, suggesting that the model incorporating CAR was superior to that incorporating CRP for prediction of lethality in mCRPC patients (**Table 3**). In addition, using multivariate model I, both CAR (HR 2.815, 95% CI 1.522–5.205, *p* = 0.0010) and TTCR (HR 0.410, 95% CI 0.215–0.784, *p* = 0.0070) were consistently found to be independent predictors for OS (**Table 3**).

**Table 2:** Univariate Cox proportional hazards analysis findings for overall survival rate after castration resistance.

Covariates	HR (95% CI)	*p*-value
Age at mCRPC diagnosis (≥80 years)	2.135 (1.072–4.252)	0.0309
Body mass index (≥22.3 kg/m^2^)	0.786 (0.438–1.412)	0.4213
ECOG PS (≥1)	2.318 (1.288–4.174)	0.0051
Hemoglobin (≥12.4 g/dl)	0.369 (0.205–0.663)	0.0001
White blood cell (≥6,100×10^9^/L)	1.529 (0.880–2.655)	0.1316
Lactate dehydrogenase (>222 U/L)	1.315 (0.756–2.290)	0.3324
Alkaline phosphatase (>266 U/L)	1.733 (0.986–3.044)	0.0588
Total protein (>7.4 g/dl)	1.138 (0.656–1.977)	0.6451
Albumin (>4.1 g/dl)	0.588 (0.331–1.045)	0.0701
CRP (>1.0 mg/L)	2.459 (1.405–4.304)	0.0016
CAR (>0.48)	3.147 (1.768–5.602)	<0.0001
PSA levels at PC diagnosis (>188.0 ng/ml)	0.653 (0.372–1.147)	0.1378
PSA levels at mCRPC diagnosis (>9.5 ng/ml)	1.409 (0.801–2.480)	0.2338
Clinical T stage (T4)	0.879 (0.467–1.655)	0.6890
Gleason score (≥9)	1.439 (0.823–2.516)	0.2017
Bone metastasis (≥4)	1.649 (0.860–3.164)	0.1323
Regional lymph node metastasis	1.271 (0.729–2.216)	0.3973
Visceral metastasis	1.197 (0.646–2.216)	0.5677
Time to castration resistance (≥12 months)	0.416 (0.230–0.750)	0.0036
First-line treatment for mCRPC (ARAT)	1.028 (0.552–1.914)	0.9318
Implementation of ARAT during treatment period (yes)	0.671 (0.264–1.706)	0.4020
Implementation of docetaxel treatment during treatment period (yes)	1.141 (0.646–2.016)	0.6483

HR, hazard ratio; CRPC, castration-resistant prostate cancer; ECOG PS, Eastern-Cooperative Oncology-Group Performance-Status Scale; CRP; C-reactive protein; CAR, CRP/albumin ratio; PSA, prostate-specific antigen; ARAT, androgen receptor axis-targeted therapy.

**Table 3:** Differences in C-index between two models containing CAR (CRP/Alb ratio) or CRP using multivariate Cox proportional hazards model.

Variables	HR (95% CI)	*p*-value	C-index
Model I			0.757
CAR (>0.48)	2.815 (1.522–5.205)	0.0010	
Time to castration resistance (≥12 months)	0.410 (0.215–0.784)	0.0070	
ECOG PS (≥1)	1.895 (0.989–3.629)	0.0539	
Age at mCRPC diagnosis (≥80 years)	1.552 (0.713–3.377)	0.2682	
Hemoglobin (≥12.4 g/dl)	0.595 (0.311–1.137)	0.1158	
Model II			0.746
CRP (>1.0 mg/L)	2.315 (1.297–4.134)	0.0045	
Time to castration resistance (≥12 months)	0.467 (0.247–0.882)	0.0190	
ECOG PS (≥1)	1.985 (1.032–3.817)	0.0400	
Age at mCRPC diagnosis (≥80 years)	1.530 (0.699–3.348)	0.2875	
Hemoglobin (≥12.4 g/dl)	0.475 (0.254–0.889)	0.0199	

HR, hazard ratio; C-index, concordance index; CRP, C-reactive protein; CAR, CRP/albumin ratio; ECOG PS, Eastern Cooperative Oncology Group Performance-Status Scale; mCRPC, metastatic castration-resistant prostate cancer.

Next, whether the combination of CAR and TTCR could be used to predict mCRPC patient prognosis with greater accuracy was assessed. The cohort was divided into three groups (0, 1, and 2 factors) based on the presence of CAR (>0.48) and/or TTCR (<12 months) (**Table 4**). Significant differences among the groups were found for several blood factors, including hemoglobin, white blood cells, CRP, and albumin. The presence of regional lymph node metastasis, visceral metastasis, and high tumor burden was also significantly correlated with number of factors present, while GS was found to have an inverse association. Further stratification using the combination of CAR and TTCR identified a stepwise reduction in both OS and PSA progression-free survival probabilities, with the shortest period found in the high CAR group with TTCR <12 months (2 factors), while the low CAR group with TTCR ≥12 months (0 factors) had the longest period (**Figures 3A, B**).

**Table 4:** Clinicopathologic features of patients divided into three groups using CAR (CRP/Alb ratio) and TTCR (time to castration resistance) risk numbers.

Characteristics	0 factors	1 factor	2 factors	*p*-value
	*N* = 40	*N* = 42	*N* = 16	
Age at mCRPC diagnosis, years	74.9 ± 9.2	76.4 ± 6.9	73.2 ± 12.0	0.2235
Body mass index, kg/m^2^	21.8 ± 3.1	22.7 ± 3.8	22.6 ± 3.8	0.5043
ECOG PS				0.0842
0	20 (50.0)	20 (47.6)	3 (18.8)	
≥1	20 (50.0)	22 (52.4)	13 (81.2)	
Serum markers at initial PC diagnosis				
PSA levels, ng/ml	203.1 (31.7–755.3)	104.1 (23.9–425.3)	268.0 (56.2–485.5)	0.543
Serum markers at mCRPC diagnosis				
PSA levels, ng/ml	8.0 (2.1–17.4)	11.7 (2.8–26.8)	23.0 (3.7–51.5)	0.1144
Hemoglobin, g/dl	13.2 ± 1.3	12.3 ± 1.8	10.9 ± 1.7	<0.0001
White blood cell, ×10^9^/L	5.6 ± 1.6	6.1 ± 1.7	7.2 ± 2.8	0.0209
Lactate dehydrogenase, U/L	222 (192–266)	212 (198–252)	243 (208–289)	0.1462
Alkaline phosphatase, U/L	239 (204–333)	264 (202–405)	409 (227–574)	0.1348
Total protein, g/dl	7.4 ± 0.4	7.4 ± 0.7	7.3 ± 0.6	0.8166
Albumin, g/dl	4.2 ± 0.4	4.1 ± 0.5	3.6 ± 0.7	0.0004
CRP, mg/L	0 (0–1.0)	1.0 (0–4.5)	9.0 (4.0–19.0)	<0.0001
CAR	0 (0–0.23)	0.26 (0.2–1.2)	2.1 (0.9–5.9)	<0.0001
Clinical T stage				0.5998
≤T3	34 (85.0)	32 (76.2)	13 (81.2)	
T4	6 (15.0)	10 (23.8)	3 (18.8)	
Gleason score				0.0093
≤8	12 (30.0)	24 (57.1)	11 (68.8)	
≥9	28 (70.0)	18 (42.9)	5 (31.2)	
Regional lymph node metastasis	13 (32.5)	23 (54.8)	12 (75.0)	0.0098
Distant metastatic site				
Bone (total)	36 (90.0)	36 (85.7)	16 (100)	0.2748
Bone (≥4)	24 (60.0)	29 (69.0)	14 (87.5)	0.1345
ny viscera (lung, liver, muscle)	8 (20.0)	9 (21.4)	8 (50.0)	0.0484
Tumor burden at PC diagnosis (CHAARTED)				0.0272
High	25 (62.5)	34 (81.0)	15 (93.8)	
Low	15 (37.5)	8 (19.0)	1 (6.2)	
Time to castration resistance				<0.0001
<12 months	0 (0)	26 (61.9)	16 (100)	
≥12 months	40 (100)	16 (381)	0 (0)	
First-line treatment for mCRPC				0.6317
ARAT	23 (57.5)	20 (47.6)	7 (43.8)	
First-generation AA	14 (35.0)	17 (40.5)	6 (37.5)	
Docetaxel	2 (5.0)	5 (11.9)	2 (12.5)	
Radium-223	1 (2.5)	0 (0)	1 (6.2)	
Implementation of ARAT during treatment period	38 (95.0)	38 (90.5)	14 (87.5)	0.5948
Implementation of docetaxel during treatment period	13 (32.5)	22 (52.4)	7 (43.8)	0.1908

Data are presented as median (interquartile range), mean ± standard deviation, or number (percentage). mCRPC, metastatic castration-resistant prostate cancer; ECOG PS, Eastern Cooperative Oncology Group Performance Status Scale; CRP, C-reactive protein; CAR, CRP/albumin ratio; PSA, prostate-specific antigen; ARAT, androgen receptor axis-targeted treatment; AA; antiandrogens.

In addition, an OS prediction nomogram incorporating CAR, TTCR, age, ECOG PS, and hemoglobin level, shown to be candidate factors in univariate analysis, was developed. This nomogram composed of five factors also had good OS predictive ability. However, its use did not improve prognostic predictive power as compared to models that used only CAR and TTCR. Details regarding this nomogram are provided as **Supplementary Figure 1**.

### Effects and prognosis for each first-line treatment method

**Figure 4:**
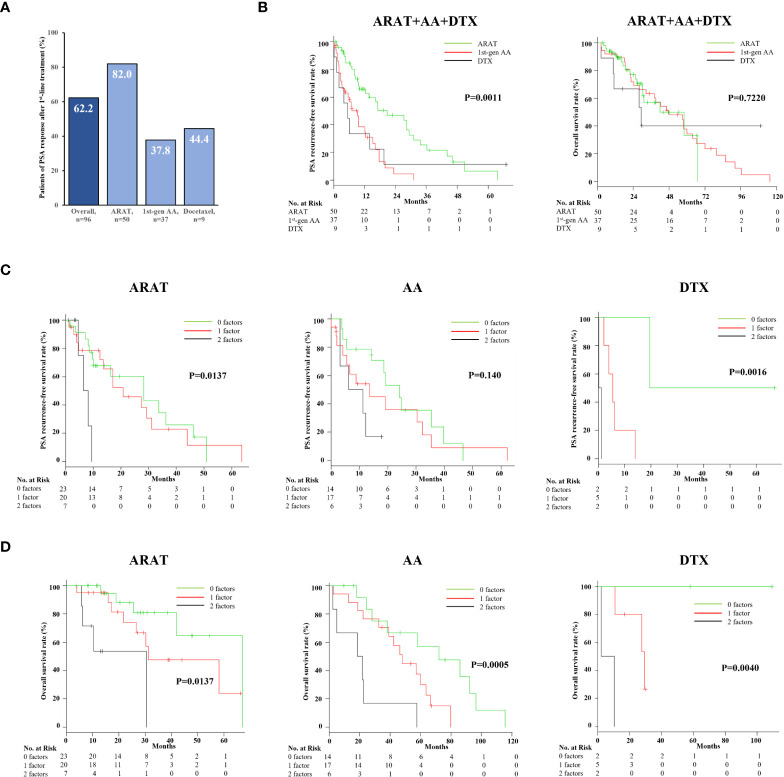
Efficacy and impact on overall survival and PSA progression-free survival of different first-line agents for mCRPC. **(A)** PSA responses for patients with ARAT, AA, and DTX treatment were 82.0%, 37.8%, and 34%, respectively. **(B)** The PSA progression-free survival rate was significantly better in ARAT patients, whereas OS was not significantly different among the three treatments. **(C)** Kaplan–Meier curve showing PSA progression-free rate after first-line treatment for mCRPC with the three treatments. The duration of PSA response in ARAT- and DTX-treated patients was significantly different among the three groups classified by CAR and TTCR. **(D)** Kaplan–Meier curve showing OS after first-line treatment for mCRPC with the three treatments. CAR- and CRP-based risk categorization effectively stratified the respective OS of mCRPC patients treated with the three different agents.

Finally, first-line treatment effects and prognosis of 96 mCRPC patients, after excluding two treated with Ra-223, were evaluated. PSA response was achieved in 62.2% overall, with ARAT having the highest rate of 82.0% among the three treatments (**Figure 4A**). Furthermore, patients who received ARAT as first-line therapy had a significantly longer time to PSA progression than those treated with DTX or AA (**Figure 4B**, *p* = 0.0011), while OS was not significantly different among the treatments (**Figure 4B**, *p* = 0.7220). Analysis of Kaplan–Meier curves showed risk stratification according to CAR and TTCR number useful for classifying PSA recurrence-free survival probability associated with each treatment, though statistical significance was not reached for patients treated with AA (**Figure 4C**). Furthermore, this risk stratification model was found to effectively stratify mCRPC patients treated with each treatment in terms of OS (**Figure 4D**).

## Discussion

We speculated that CAR and TTCR reflect mCRPC patient prognosis, and their use in combination could be useful for prognostic prediction. A retrospective investigation of mCRPC patients treated at our institution was performed with noteworthy findings obtained, as detailed in the following.

mCRPC patients with CAR greater than 0.48 had significantly shorter survival and duration of PSA response after initial treatment as compared with those with lower CAR. Notably, CAR remained an important prognostic factor for OS even in multivariate analysis that incorporated various patient and tumor factors. These findings are consistent with previous studies of castration-resistant PC patients (16, 17), especially that presented by Uchimoto et al. (17), which noted an optimal CAR cutoff value of 0.50, nearly the same as in the present study.

Chronic inflammation is closely related to cancer progression; thus, attention has focused on the relationship between elevated CRP and prognosis in cancer patients including PC. A prospective population-based cohort study conducted by Stikbakke et al. showed that elevated serum CRP levels had adverse effects on PC risk and prognosis (24). Also, two studies that employed meta-analyses of data obtained from previous reports confirmed CRP as an effective predictor of poor outcome in PC cases including mCRPC (25, 26). Interestingly, one of these (26) showed that low albumin was also a significant factor associated with poor prognosis in mCRPC patients. Decreased albumin leads to increased CRP through release of various cytokines, indicating a negative correlation between these factors (10). Furthermore, changes in CAR, composed of CRP and albumin, may be more sensitive to patient and/or cancer conditions than CRP or albumin alone. Indeed, the present findings showed that multivariate models incorporating CAR more accurately predicted OS in patients with mCRPC than models incorporating CRP. This superiority of CAR over CRP or albumin for predicting mCRPC patient prognosis was also confirmed by Uchimoto et al. (17).

TTCR was also confirmed as an independent predictor of OS after mCRPC development. Patients with a TTCR of ≥12 months had a median OS of 30 months, whereas those with a TTCR of <12 months was significantly shorter (20.7 months). This trend was also found for the period until PSA progression. Although some studies failed to identify OS differences between TTCR subgroups after castration resistance was acquired (18, 20), these findings show a clear prognostic difference based on TTCR classification, as previously reported (17, 19, 27). Importantly, use of 12 months for prognostic definition by TTCR was also adopted in studies of PC patients in Japan treated with ADT who acquired castration resistance, while Miyake et al. further classified TTCR and reported that those with ≤6 months had the worst prognosis (17–19, 27). A study that divided mHSPC patients into those who received ADT+ARAT or DTX also showed that TTCR <12 months strongly associated with poor prognosis (20). Therefore, TTCR <12 months seems accurate for predicting worse OS even in this combination therapy era.

Recently, studies have analyzed changes induced in mHSPC by hormone therapy at the genetic level, with interesting results obtained. Zurita et al. showed that amplification of *AR* and *MYC*, or loss of *TP53* and *RB1*, known as poor prognostic factors, was enhanced after hormone therapy resistance (28). Also, genome-wide loss-of-heterozygosity (gLOH), a genomic instability marker, was increased with emerging resistance to hormonal therapy, while higher gLOH was closely associated with the presence of altered homologous recombination-repair (HRR) genes (*BRCA2*, *PALB2*, and *FANCA*). Kimura et al. reported that mHSPC patients with germline HRR mutations including *BRCA2* and *PALB2* had significantly shorter TTCR (29). Thus, it is considered that shorter TTCR reflects, at least in part, genetic differences or mutations in the host or tumors.

Finally, prediction of OS and time to PSA progression was confirmed possible by dividing mCRPC patients into three groups according to values for CAR (>0.48) and TTCR (<12 months), identified as poor prognostic factors in this study. Furthermore, the combined classification of CAR and TTCR was able to predict duration of response and prognosis associated with each first-line mCRPC treatment. These observations are not surprising, as use of these factors combined involves differences in a variety of host- and tumor-side poor prognostic factors, such as low PS, anemia, high tumor stage, and metastasis. Previous results indicating CAR or TTCR ability to predict treatment outcome in mCRPC patients also support our findings. Specifically, Uchimoto et al. reported that prognosis of patients with high CAR was poor regardless of ARAT, AA, or DTX treatment (17). Gültürk et al. showed that DTX-treated mCRPC patients with TTCR <12 months had significantly shorter durations of response and OS than those with TTCR >12 months (30). Thus, we concluded that classification of mCRPC patients based on both CAR and TTCR enables accurate predictions of patient prognosis as well as efficacy of each therapy.

This study has several limitations, including retrospective design and low number of mCRPC patients treated at a single hospital. Owing to the small sample size, the CAR and TTCR cutoff thresholds used may not be adequate to reflect prognosis in other cohorts. However, several previous studies have used prognostic cutoff values close to those defined in the present study for both CAR and TTCR. Furthermore, patients who started initial treatment for mCRPC before ARAT was introduced in Japan were also included. Selection bias may exist regarding treatment options, since therapy choice for individual patients might have been based on disease severity. Also, patients who received combination therapy in a castration-sensitive stage or did not have distant metastasis at the time of castration resistance did not receive focus. Finally, exclusion of other candidate blood biomarkers, including neutrophil–lymphocyte ratio and inflammatory line interleukin, is another limitation. For example, it has been pointed out that pivotal inflammatory cytokines that are members of the interleukin-1 family may serve as important biomarkers for predicting clinical stage and prognosis in patients with PC (31). Prospective studies with larger populations that overcome these limitations are required to validate and confirm our findings.

## Conclusion

CAR and TTCR were found to be independent predictors of prognosis and treatment response in mCRPC patients. In addition, prognosis after mCRPC development and therapeutic efficacy of treatment options may be predicted more accurately by combining CAR and TTCR. It is considered that this method can accurately identify patients who may benefit from treatment and also provide useful information regarding optimal treatment. Future large-scale prospective studies will be necessary to confirm the present preliminary findings and may lead to development of effective risk models.

## Data availability statement

The raw data supporting the conclusions of this article will be made available by the authors, without undue reservation.

## Ethics statement

The studies involving human participants were reviewed and approved by Ethics Committee of Toho University Omori Medical Center. Written informed consent for participation was not required for this study in accordance with the national legislation and the institutional requirements.

## Author contributions

YM, KNag, and KNak contributed to conception and design of the study. FY, SH, MU, and HA collected patient data. YM wrote the first draft of the manuscript. KS performed the statistical analysis. All authors contributed to the article and approved the submitted version.
