# Supplementary material for: Combination of C-reactive protein/albumin ratio and time to castration resistance enhances prediction of prognosis for patients with metastatic castration-resistant prostate cancer
Source: Front Oncol. 2023 Jun 2;13:1162820. doi: 10.3389/fonc.2023.1162820 (PMC10272398; doi:10.3389/fonc.2023.1162820)

**Supplemental Figure 1. Constructed prognostic nomogram and evaluation using Kaplan-Meier curve.**

**A**. Nomogram for predicting one- and two-year overall survival of patients with metastatic castration resistant prostate cancer among the total cohort. Age >80 years corresponds to 42 points, PS>1 to 62 points, TTCR<12 months to 85 points, Hgb<12.4 to 50 points, and CAR>0.48 to 100 points. **B**. The cutoff value was determined by grouping the patients evenly into three subgroups after sorting by total score (0–99, 100–249, >250), with each group representing distinct prognosis based on higher score and worse prognosis.


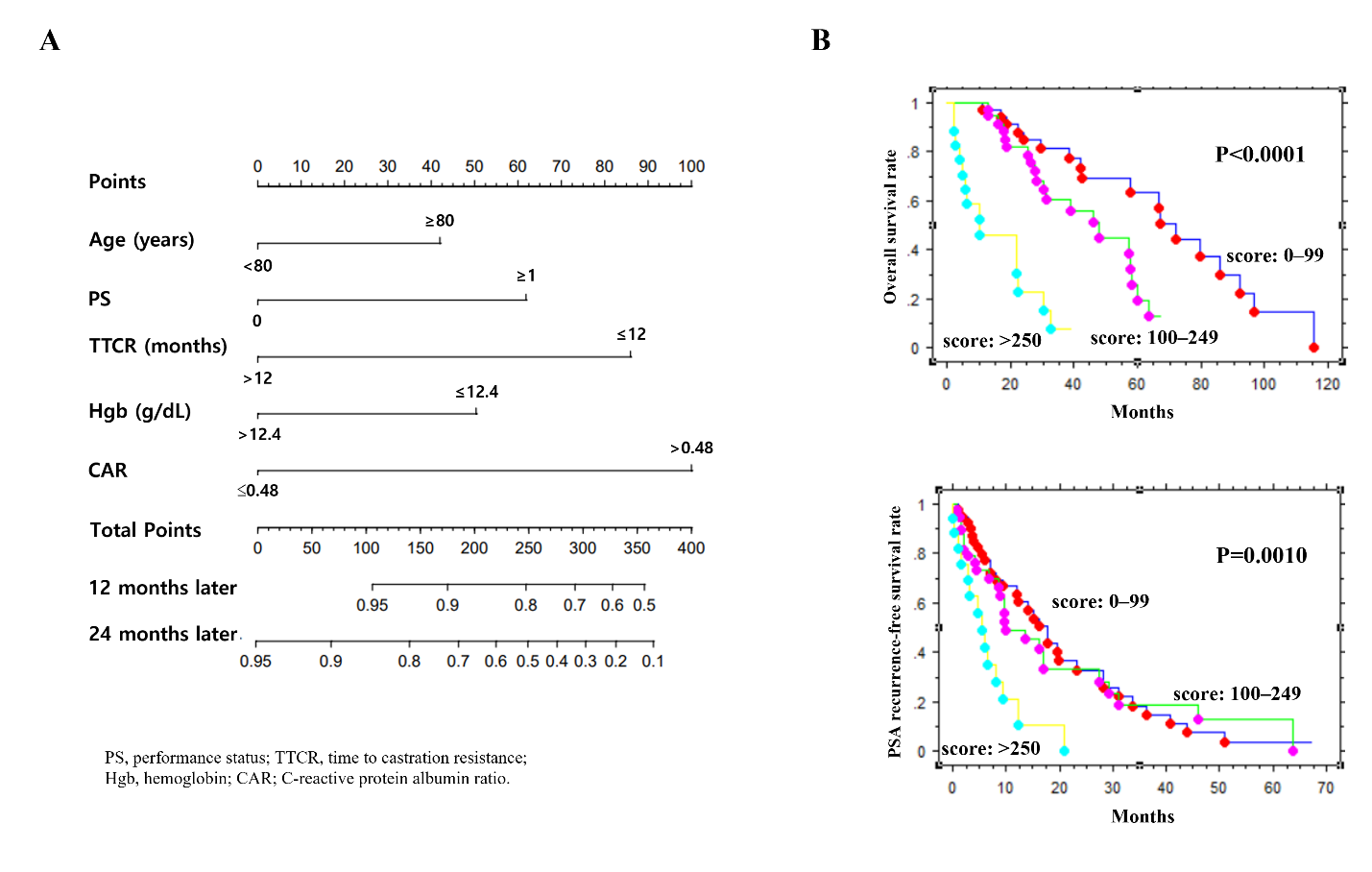

Supplement: Supplementary file 1 [file DataSheet_1.docx]
